# Efficacy of Second-Look Ultrasound with MR Coregistration for Evaluating Additional Enhancing Lesions of the Breast: Review of the Literature

**DOI:** 10.1155/2018/3896946

**Published:** 2018-10-21

**Authors:** Maria Antonietta Mazzei, Letizia Di Giacomo, Alfonso Fausto, Francesco Gentili, Francesco Giuseppe Mazzei, Luca Volterrani

**Affiliations:** ^1^Department of Medical, Surgical and Neuro Sciences, Diagnostic Imaging, University of Siena, Azienda Ospedaliera Universitaria Senese, Viale Bracci 10, 53100 Siena, Italy; ^2^Diagnostic Imaging, Azienda Ospedaliera Universitaria Senese, Viale Bracci 10, 53100 Siena, Italy

## Abstract

Contrast enhanced magnetic resonance imaging (CE-MRI) has acquired a central role in the field of diagnosis and evaluation of breast cancer due to its high sensitivity; on the other hand, MRI has shown a variable specificity because of the wide overlap between the imaging features of benign and malignant lesions. Therefore, when an additional breast lesion is identified at CE-MRI, a second look with targeted US is generally performed because it provides additional information to further characterise the target lesion and makes it possible to perform US-guided biopsies which are costless and more comfortable for patients compared with MRI-guided ones. Nevertheless, there is not always a correspondence between CE-MR findings and targeted US due to several factors including different operator's experience and position of patients. A new technique has recently been developed in order to overcome these limitations: US with MR coregistration, which can synchronise a sonography image and the MR image with multiplanar reconstruction (MPR) of the same section in real time. The aim of our study is to review the literature concerning the second look performed with this emerging and promising technique, showing both advantages and limitations in comparison with conventional targeted US.

## 1. Introduction

Contrast-enhanced magnetic resonance imaging (CE-MRI) of the breast has progressively acquired a central role in the field of detection and evaluation of breast cancer due to its high sensitivity, ranging from 94% to 100% for invasive carcinoma and from 40% to 100% for ductal carcinoma in situ (DCIS) [1–5]. On the other hand, MRI still shows low to moderate specificity (72%) and moderate positive predictive values (PVVs) for lesion characterisation [6] due to a wide overlap between the imaging features of benign and malignant lesions [7–15]. Therefore, when abnormalities detected on MRI are occult on mammograms or are not identified with previously performed breast ultrasonography (US), a targeted second-look US is commonly prescribed [16]. The use of targeted US has several advantages: first it can provide additional information for a further characterisation of the additional lesion when correlated with MRI findings [17, 18]; second, it makes it possible to practise US-guided biopsies which are preferable to the MR-guided ones because they are superior in terms of accessibility, efficacy, and comfort for patients [19]. However, it has been observed that the detection rate of the additional lesions with second-look US is variable, with a reported range between 23% and 82,1%; this wide range of variability can be attributed to different factors which may include technical differences and different reader experience [20]. A new innovative technique has recently been developed in order to overcome this problem by using MR coregistration during live US examination. Different vendors have used various names for this revolutionary technology which enables coregistration of a previously acquired MR volume during US examination with magnetic sensors on the US probe and a transmitter connected to US equipment [21, 22]. Several studies have shown that MR navigated US is an accurate method which increases US detection rate of MR-detected additional lesions [21–32]. The aim of our study was to give an update of the literature concerning the utility of second-look US coregistered with breast MR showing both the advantages and disadvantages of this emerging and promising technique.

## 2. Materials and Methods

We performed computerised research on PubMed database, Google, and ResearchGate by using the following search terms: “Breast volume navigation”, “second-look breast lesions” and “second-look real time-ultrasonography”. Full texts where then retrieved, including those of reviews concerning various coregistration techniques; nevertheless, in three cases it was not possible to obtain either the full text or the abstract because they were available only in Japanese language. Finally, in order to make our research more complete, a systematic review of the references of each article was performed. After carefully reading the articles, we analysed the methods and the systematic errors of each system if reported, and compared the clinical value of each reported technology.

### 2.1. Technical Principles of Ultrasound with MR Coregistration

**Figure 1:**
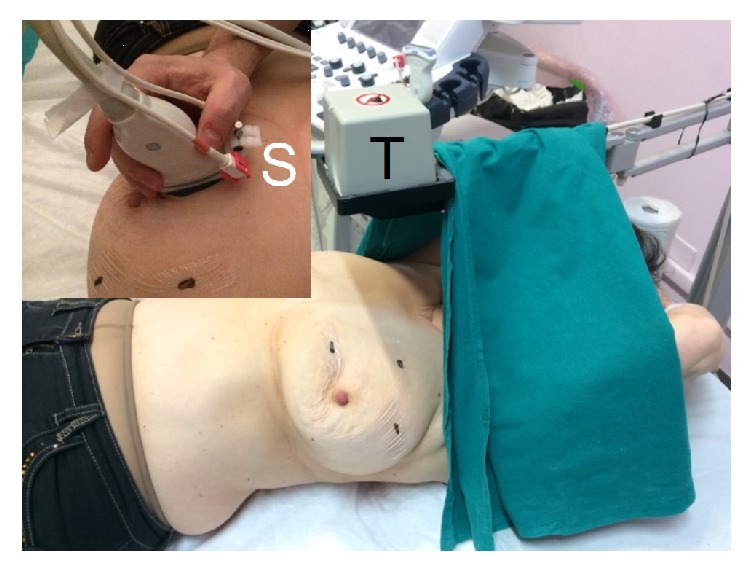
US-MR coregistration equipment composition: a pair of freehand sensors (S) and a fixed transmitter (T) connected to a position-sensing unit embedded in the US equipment.

The equipment consists of two electromagnetic sensors which are attached to the US probe, a portable electromagnetic transmitter that is positioned near the patient under examination, and a position-sensing unit that connects the electromagnetic sensors and the transmitter enabling the tracking probe position and orientation within the electromagnetic field embedded in US equipment. After uploading the preacquired MR volume in the US equipment, coregistration can be obtained by matching skin and MR markers. The matching can be obtained coupling at least three pairs of points, one point and a plane, or automatically, according to different vendors. The coregistration is usually displayed on a US monitor showing US and MR images side by side or overlaying both images, the so-called fusion images (Figure 1). According to a recent review of Young Park et al. the following ultrasound navigation systems are commercially available: Real-time Virtual Sonography (Hitachi Medical Corporation); Volume Navigation (GE Healthcare); eSie Fusion (Siemens Healthcare); Virtual Navigator (Esaote); PercuNav (Philips Healthcare); and Smart Fusion (Toshiba Medical Systems Corporation). These systems operate on the basis of similar equipment components and technical principles as described above [33]. The possibility of synchronising MR and US images by using multiplanar reconstruction (MPR) of the same section in real time is a great advantage, as it makes the exam more objective and less operator-dependent.

Nevertheless, an important limitation which has to be taken into account is that this new technique requires images information obtained from two different modalities at different times; breast tissues are soft and easily deformable, so that the position of the different structures may undergo significant variations from one exam to another, causing spatial displacement and misalignment. In order to overcome this problem, it is necessary to perform a nonrigid registration which requires application of the best transformation algorithm, making it possible to obtain an alignment with the least error between two breast images [34, 35]. Different coregistration methods have been developed with the aim of obtaining the best result. At one extreme, patient position and algorithms have been developed to reduce deformations due to the mechanical properties of the breast as much as possible; at the other extreme, algorithms have been developed to model the deformations imposed on the images using simple functions. In this case, landmarks are identified between the two images to be registered and a transformation is computed to coregister these landmarks. With regard to the breast, anatomic features can be either at the surface or internal [36].

### 2.2. Additional Supine MRI for Volume Navigation System: Technical Limits and Advantages

Breast MRI is commonly performed in the prone position because it minimises breast motion due to respiration and reduces the potential interference with the beating heart. In addition, the coil coupling is improved. [37]

Nevertheless, breast tissue is highly mobile and deformable and composition may vary with the individual hormonal status such as menstrual cycle. These factors may cause difficulties in coregistration due to the different position between ultrasound and MR examination (supine versus prone) that may lead to a misdiagnosis of breast lesions on second-look ultrasound [33]. In an attempt to minimise spatial displacement, various solutions have then been adopted.

Piron et al. developed a hybrid biopsy system based on the standard closed-bore MR magnet configuration, merging prebiopsy MR and real-time US information in one procedure, and proposed to perform both US and MR image acquisition in the prone position, obtaining encouraging results. [38].

In a pilot study, Causer et al. evaluated the accuracy of the same MR-US coregistration system in vivo; both MR and US examinations were performed with the patient in the prone position using a system designed at their institution that featured a redesign of the MR bed and coil system with added computer software assistance for calculating ultrasound transducer placement; the mean* x*,* y*, and* z *plane errors for displaying MR additional lesion with US were 2.5 mm (range, 0.9-6.3 mm), 1.1 mm (range, 0-4 mm), and –2.6 mm (range -0.9 to 5.3 mm), respectively, with no significant clinical difference. Moreover, after applying the correction value to the initially calculated error measurement on the z-plane, the error decreased to −1.7 mm (range, −0.04 to 4.2 mm) [39]. However, in these cases the position of the radiologist was below the patient during the US examination and interventional procedures and it seems to be less practical in clinical routine or during a biopsy procedure [25]. In a recent study, Young DK et al. reported a median difference in lesion-to-nipple distance on supine and prone MRI of 8 mm (0-34 mm) in the horizontal direction and 5 mm (0-39,5 mm) in the vertical direction; in addition, thirteen lesions had a difference greater than 1 cm in both horizontal and vertical directions. No significant differences were found in both directions with respect to upper and lower locations [29].

**Figure 2:**
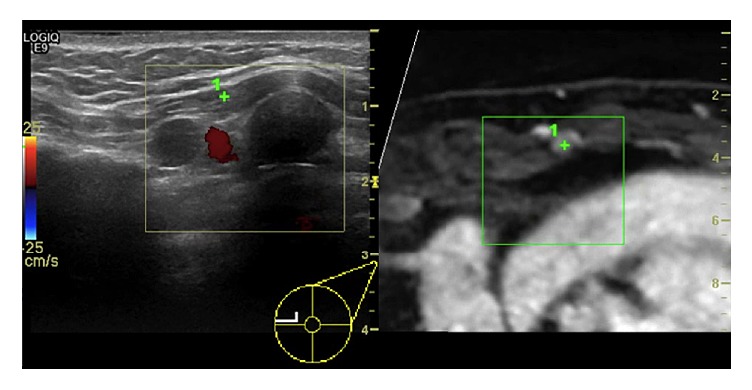
Ultrasound image (left side) with the corresponding multiplanar reconstructed MR image (right side) of a 55-year-old woman who underwent a previous surgery of the left breast for invasive ductal carcinoma (IDC, pT2N0). After 6 months, a follow-up MR was performed showing a rounded enhancing lesion in the left internal mammary chain (green cross). Second-look ultrasound with coregistration revealed a pathological lymph node.

**Figure 3:**
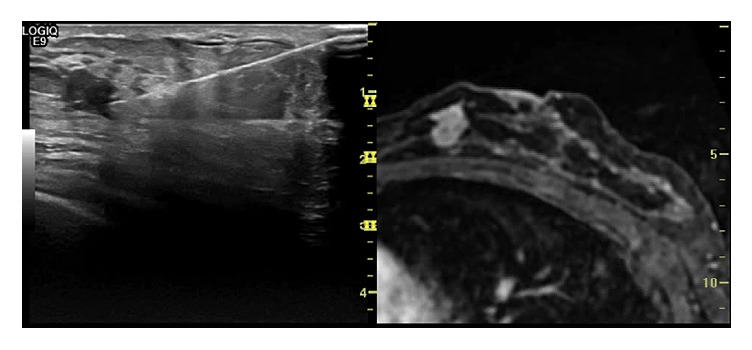
A 48-year-old woman with a previous left breast quadrantectomy (lower outer quadrant) for an invasive ductal carcinoma (IDC, pT1N0) underwent a MR follow-up 5 months after surgery that showed an additional enhancing lesion at the confluence of the inner quadrants near the nipple. Second-look ultrasound with MR coregistration confirmed the lesion which was biopsied by fine needle aspiration; the histological finding revealed an IDC.

Fausto and coworkers found good accuracy and reproducibility of volume navigation by combining US and MR images which had been both acquired in the supine position; in particular, MR was acquired in the supine position, with upper extremities extended over the head using a double synergy body coil with sensitivity encoding, covering both breasts. Breast compression was minimised using a dedicated mattress and two straps. Live US exams were performed in healthy volunteers in the supine position using a platform configured with volume navigation technique (LOGIQ E9, GE Healthcare) and a 6–15 MHz transducer with a geometry, which allows the visualisation of a wide field-of-view in both conventional and trapezoid imaging [25, 26]. The latter findings have subsequently been confirmed in another study, again conducted by Fausto and coworkers using the same technique described above, which showed that the use of second-look ultrasound with volume navigation makes it possible to objectively correlate MRI additional lesions with ultrasound appearances, showing a significant higher detection rate in comparison with conventional targeted US but without differences in the number of false positive or true positive lesions [24]. Moreover, Nakano and coworkers, who were the first to quantify the positioning error of a magnetic navigation system in breast imaging by performing MRI in the supine position, reported an overall 3D mean positioning error of approximately 12 mm, which is clinically acceptable [22]. Therefore, in light of these considerations, we can affirm that although an additional supine MR examination can be time-consuming [33], requires the use of more contrast, and reduces image quality, it has the major advantage of better correspondence with standard US and surgical position that is very helpful for both targeting and biopsy [23, 36] (Figures 2 and 3).

### 2.3. Accuracy and Feasibility of Second-Look with MR Coregistration: A Comparison with Conventional Targeted US

Different breast-imaging modalities offer complementary information that can help to establish a diagnosis or assist the clinician for a therapeutic gesture [34].

In particular, the advantages of incorporating ultrasound in image fusion consist in the real-time images (which enable image-guided intervention), the lack of radiation to both patient and staff, and the possibility of comparing findings between different modalities [40].

In our research, we found 11 original papers evaluating the diagnostic performance of US-MR coregistration, published from October 2008 to October 2017, which showed that this technique may identify additional enhancing lesions with high accuracy. The first of these was a pilot study by Causer and coworkers, which was carried out to determine the accuracy of MR-US coregistration system in vivo for showing breast lesions visible on MRI and US. Both techniques were performed in the prone position, lesion pathology was determined on the basis of imaging features for cysts or histopathology for masses, and targeted lesions were displayed on the US monitor on the basis of transducer coordinates calculated from MR images. By using these methods, they found that mean lesion size correlated well (*R *=0.99) on MR (11.4 mm; range, 6–28 mm) compared with US (10.3 mm; range, 6–28 mm) and mean error measurement on the three planes was clinically acceptable. Although results were encouraging, the small number of lesions included in the study (13) was an important limitation [39].

With regard to the other 10 more recent studies, the number of patients enrolled ranged from a minimum of 51 [22] to a maximum of 831 [29] and MRI was performed either for staging a known breast cancer only or for both staging and solving diagnostic problems. MR for coregistration was always performed in the supine position and in 6 out of 10 cases was obtained on 1.5 T equipment [21–23, 27, 29, 32] while in the other 4 on 3 T equipment [24, 28, 30, 31]. In 5 out of 10 studies all the detected additional lesions were studied with second-look US with and without MR coregistration and in one case [21] it was specified that patients had been studied with mammography, US, and MR in addition to coregistration (Table 1).

The reported detection rates of second-look with conventional US were highly variable, ranging from 30% to 61.2%, while those concerning MR coregistration were much higher, ranging from 83% to 95,5%; moreover, all enhancing lesions that were detected at second look with conventional US could be identified by using the coregistration system. Shogo Nakano et al. also showed that the overall sensitivity for detecting index tumours was 85% for mammography, 91% for US, 97% for MR, and 98% for the coregistration system (100% invasive ductal carcinomas, 100% mucinous carcinoma, and 88% ductal carcinomas in situ); notably, in one instance in which the cancer was not seen on MR, US-MR coregistration detected it with the supplementation of sonography [21].

In the other 5 studies, US and MR coregistration was only performed with the aim of identifying the MR-additional lesions not found at second look with conventional US; the reported values concerning the detection rate of US alone were in line with the previous ones and coregistration was successful in detecting US-missed additional lesions in a high percentage of cases (detection rate from 78% to 100%) (Table 2).

Accordingly with previous studies, Elena Pastor Pons found that diagnostic performance of US-MR coregistration for identifying malignant nodules, considering overall lesions and the subgroup of ILSM, was sensitivity 96.3% and 100%, specificity 18.8% and 30.7%, positive predictive value 66.7% and 43.7%, and negative predictive value 75% and 100%, respectively; in addition, US-MR coregistration enabled biopsy of 2 metastatic lymph nodes [27].

All authors reported high rates of histological confirmation of target lesions obtained under sonography guidance, showing that US-MR coregistration is a feasible alternative to MR-guided biopsy which is time-consuming, expensive, and not widely available [17]. In particular, an important result which emerged in a recent study of Aribal et al. was that pathologic diagnoses of all malignant and high risk lesions were achieved by ultrasound guided biopsy using US-MR coregistration technique [30].

Moreover, 2 studies reported that the few added lesions with no Real-time Virtual Sonography (RVS) correlate were more benign than malignant [23, 32] and Kang DK et al. found that 2 out of 4 lesions not detected on US-MR coregistration examination disappeared, while the remaining 2 did not exhibit any change on follow-up MR [29]. Although these results require further confirmation, they suggest that US-MR coregistration could help to reduce the number of false positives thus avoiding useless biopsies.

Some authors analysed the association between US, MR and histological characteristics of target lesions and US-MR coregistration results.

In only 2 out of 10 articles it was found that US-detected lesion size during US-MR coregistration alone was significantly smaller than that detected by conventional B-mode [22, 24]. Shogo Nakano et al. also reported that the mean tumour size provided by RVS and MRI-Multiplanar Reformation was 12.3 mm and 14.1 mm, respectively (r = 0.848, p < 0.001) [22]. Nevertheless, the results obtained in another 3 out of 10 articles regarding this parameter showed no statistically significant association [21, 23, 31]. Furthermore, an analysis again conducted by Shogo Nakano and coworkers, showed that, compared with the use of US alone, US-MR coregistration was useful in identifying lesions in patients whose diagnostic images exhibited smaller differences in echogenicity between the interior and exterior parts of the tumours, and which exhibited non-tumoral low-echo regions in the background [21].

In 2 out of 10 studies, statistically significant differences were found between some MR characteristics of US-MR coregistration detected lesions and those of undetected ones; in particular, Shogo Nakano found that identification by US-MR coregistration was more likely when the MR-detected lesions appeared as one or more foci (94%) or as a mass (89%) (p = 0.001, p < 0.001, respectively) than when lesions were described as showing non-mass-like enhancement (80%). Moreover he found that US-MR coregistration had a higher detection rate for lesions of 5 mm and those of 5-10 mm at MRI (p = 0.001, p <0.001, respectively) and observed that lesions detected by coregistration technique alone were more likely to be found around mammary fascia (71%), whereas those identified by conventional US were more frequently found within the mammary gland (61%) (p = 0.023) [23]. The latter findings were in line with a recent study conducted by Park et al. which found a statistically significant difference in lesion depth between the group of US-MR coregistration detected lesions and that of US detected ones; in fact lesions of the first group tended to be located in the middle or posterior portion of breast parenchyma (78.3% [18 out of 23] for coregistration vs. 46.3% [19 out of 41] for US), whereas those of the second group tended to be located in the anterior portion of parenchyma (53.7% [22 out of 41] for US vs. 21.7% [5 out of 23] for coregistration). No significant difference in detection with conventional US and coregistration techniques was found on the basis of lesion size, distance between the nipple and the lesion, lesion shape, orientation, margin, posterior features, association with calcification or duct changes, lesion type (mass-like vs. non-mass-like lesions), and kinetic curve assessment [31]. In contrast Uematsu T et al., Kang DK et al., and Watanabe R et al. found no significant correlation between MR characteristics and lesion detection with US-MR coregistration or US alone [28, 29, 32].

Interestingly, Park AY et al. also observed that lesions detected during the coregistration technique are at increased risk of malignancy compared to conventional US (McNemar test 21 vs. 11, P < .001) and after second-look US, the optimal treatment plan changed in 16 of 55 (29.1%) patients; in particular, in 9 out of 16 patients (60%) the treatment plan changed because of additionally found lesions by coregistration technique [31]. Similarly, Watanabe R et al. reported that in 7 out of 53 patients (13%) surgical management was altered by US-guided biopsy of the lesions detected by coregistration technique [32].

## 3. Discussion

As previously discussed, the sensitivity of breast MRI for the detection of breast cancer is high, but its specificity is only moderate, ranging from 37% to 100% [16]. It is then essential to biopsy suspicious MR-detected lesions to make a definitive diagnosis [22]; MR-guided breast biopsy is gradually increasing, especially when lesions are visible on MRI but not on conventional imaging [41–43]. Nevertheless, these techniques are not widely available and require the use of expensive MR magnets, time, and personnel [21]. Furthermore, the positive predictive value of MR-guided biopsy has been reported as relatively low due to the high benignancy rate found at pathology, thus leading to a high number of unnecessary biopsy procedures even in experienced settings [25].

For these reasons, second-look targeted US has become the tool of first choice to further characterise additional MR-detected lesions.

A recent meta-analysis by Spick et al., including seventeen studies, found that lesion detection rate at second-look US was very heterogeneous ranging between 22.6% and 82.1% (pooled rate, 57.5% [1266 of 2201]; 95% confidence interval [CI]: 50.0%, 64.1% [random-effects model];* I*^2^ = 90.9%;* P *< .0001). The highest second-look US detection rates were observed for mass lesions (as opposed to non-mass lesions) and for malignant (vs. benign) lesions (*P *< .001 for both). However, they also observed that if a lesion is not visualised at second-look US, malignancy might occur in a pooled estimate of 12.2%, and therefore a negative second-look US cannot exclude malignancy [20]. Similarly, a recent review of literature which analysed sixteen original papers evaluating the diagnostic performance of breast second-look ultrasound reported that this technique makes it possible to find a correlation to MR additional lesions in 64% of cases (weighted average; SD 18%), ranging from 23% to 89%, with a probability of cancer detection at second-look ultrasound ranging from 8% to 56% (weighted average of 36%) compared with an MR-guided biopsy weighted average of 21% [24]. The success of US examination depends on several factors such as the operator's experience, breast size, findings, and lesion depth; moreover, because the operator has to perform the US-guided biopsy based only on mentally visualised positional information from the MR, there is no direct evidence that the lesion has been accurately detected and biopsied [22].

In order to overcome these problems, a new coregistration technique carrying different names depending on its vendors has recently been developed, which can synchronise the sonographic and MR images during live US [21].

Our analysis showed that the US-MR coregistration technique increases the overall accuracy of second-look US due to its higher sensitivity for additional MR-detected lesions compared with conventional US; in particular, the authors reported detection rate values ranging from 83% to 100% and some of them found that RVS was successful in detecting additional lesions blinded at US in an high percentage of cases (detection rate from 60% to 100%). Another important advantage is that US-MR coregistration is an easy-to-use tool that is well-integrated in US equipment and could be a way to reduce operator-dependency of US when lesion displacement due to different position of the patient has a fundamental impact on detection [24].

In addition, although further confirmation is needed, the results obtained by some authors suggested that the US-MR coregistration technique could improve the identification of high risk and malignant lesions and could also be helpful in detecting suspicious lymph nodes [33]; this means that this technique may significantly reduce the number of MR-guided biopsies enabling operators to select cases that really require it and to choose the most appropriate treatment plan for each patient. Nevertheless, there are some technical limitations which have to be taken into account: first of all, pressure applied to the probe may alter the depth of lesions and distort anatomic landmarks depicted by MRI, especially during interventional procedures; it is thus necessary to conduct the operation gently, avoiding the application of excessive pressure on breast tissue [30]; secondly, although the US-MR coregistration technique enables the identification of deep lesions which are often missed on conventional US [23, 31], the patient has to maintain the supine position that may hide the lesions localised in the lateral portions of the breast and hinders US-guided biopsy of peripheral lesions [30].

Accurate localisation is also essential for adequate surgical removal of breast tumours, in which an optimal balance between good cosmetic results and preservation of resection margins is the primary goal [44]. Some studies have been conducted in order to investigate the feasibility of the US-MR coregistration to demarcate breast cancer. Anderliesten et al. reported that image-guided coregistration to demarcate breast cancer, on the basis of preacquired MR images, in a supine orientation, appears feasible if patient's breath is tracked during the navigation procedure, positional uncertainty is visualised, and pressure on the location instrument is released after the verification of its position [45]. Moreover, Chang et al. found that the tumour size, estimated by US-MR coregistration technique, was more strongly correlated with the histological one than with US alone; measurement of the lesions by US-MR coregistration technique was significantly more accurate for mass type lesions detected on MRI. In addition, accurate measurement of mass extent was improved with the US-MR coregistration technique, compared with US alone, in patients who had non-mass type lesions on MRI and who had undergone neoadjuvant systemic chemotherapy [44]. In light of these considerations, we could assume that if these findings will be further confirmed, US-MR coregistration technique may become an important tool not only for second-look US, but also for surgical planning.

Limitations of volume navigation technique are associated with errors in coregistration of MR dataset and live US because it is primarily based on the assumption that the structures within the data volume (i.e., the body part studied) have fixed positions, relative to each other, in the two different imaging modalities [46].

Nevertheless, as previously noted, breast tissues are soft and easily deformable so that they can undergo relevant modifications from prone to supine position, thus causing discrepancies and misalignment in coregistration. Compensation algorithms for such causes of misalignment have been proposed, but they are still limited in medical applications because of the complex physical properties of tissues [46]. Another important factor which may reduce the effectiveness of US-MR coregistration is breast hypertrophy. In patients with high breast volume, the transition from the prone to the supine position determines a large variability of tissue placement and possibility of dislocation. Using two anthropomorphic measurements as suggested by Sigurdson, a good selection of subjects can be made [47]. Actually, this method enables a precise determination of breast volume, thus helping operators to select patients who need MR-guided biopsy, especially if the additional lesion is localised at the external quadrants and far from the skin. Eventually an additional supine MRI may be used to obtain a better MR-US match of the lesion since US is performed with the patient in the supine position too [28]. Moreover the supine position allows an accurate preoperative planning since the patient is analysed in the same position adopted on the operating table [23]. However this approach has several disadvantages: first of all, image quality is lower compared with prone MRI due to respiratory or heartbeats artefacts and to the use of nondedicated coils [22, 25, 30]; secondly, an additional MRI examination in the supine position is time-consuming, requires additional administration of contrast agent, or may be unavailable [33].

On the other hand several authors did not find significant misalignment of the lesions on the three axes using standard US-MR coregistration, demonstrating that this technique is accurate and feasible even to locate lesions within a limited volume [22, 25, 29, 46].

## 4. Conclusions

In light of these considerations, we can affirm that US-MR coregistration technique is an accurate and feasible imaging technique which can significantly increase both the detection rate of additional enhancing lesions of the breast and the number of US-guided interventional procedures, which are preferable to MR-guided ones. Moreover, it is easier to perform, much less operator-dependent, and also comfortable for the patient because it does not require radiation and additional preparation. Although further studies are needed in order to confirm these findings and to overcome technical limitations, results are encouraging and suggest that US-MR coregistration technique may become an important tool for second look which could also help operators to choose the most adequate treatment plan and patient management.

## Figures and Tables

**Table 1:** Studies in which both conventional US and US with MR coregistration have been perfomed.

**Authors**	**Number of patients with MR additional lesions**	**Number of MR additional lesions**	**US-correlate**	**US with MR coregistration correlate**
S.Nakano 2009	17	23	7/23 (30%)	19/23 (83%)
S.Nakano 2012	51	63	42/63 (67%)	63/63 (100%)
S.Nakano 2012	55	67	18/67 (30%)	60/67 (90%)
E.P Pons 2014	148	28	3/28 (11%)	21/28 (75%)
A.Y.Park 2017	70	67	41/67(61,2%)	64/67 (95,5%)

**Table 2:** Studies in which US with MR coregistration has been only performed in order to identify US-missed MR additional lesions.

**Authors **	**Number of patients with MR additional lesions**	**Number of MR additional lesions**	**US correlate**	**US- missed additional lesions detected with US with MR coregistration**
A. Fausto 2012	129	207	83/207 (40%)	124/124 (100%)
T. Uematsu 2016	70	78	50/78 (64%)	24/28 (85,7%)
D.K. Kang 2017	101	119	79/119 (66,4%)	31/40 (78%)
R. Watanabe 2017	53	59	20/59 (34%)	33/39 (85%)
E. Aribal 2017	73	77	51/77 (66%)	26/26 (100%)

## References

[1] Orel S. G., Schnall M. D. (2001). MR imaging of the breast for the detection, diagnosis, and staging of breast cancer. *Radiology*.

[2] Harms S. E., Flamig D. P. (2001). Breast MRI. *Clinical Imaging*.

[3] Berg W. A. (2001). Overview of breast imaging. *Seminars in Roentgenology*.

[4] Orel S. G. (2001). MR imaging of the breast. *Magnetic Resonance Imaging Clinics of North America*.

[5] Kuhl C. K. (2007). Current status of breast MR imaging: Part 2. Clinical applications. *Radiology*.

[6] Menezes G. L. G., Knuttel F. M., Stehouwer B. L., Pijnappel R. M., Van Den Bosch M. A. A. J. (2014). Magnetic resonance imaging in breast cancer: A literature review and future perspectives. *World Journal of Clinical Oncology*.

[7] Tilanus-Linthorst M. M., Obdeijn I. M., Bartels K. C., de Koning H. J., Oudkerk M. (2000). First experiences in screening women at high risk for breast cancer with MR imaging. *Breast Cancer Research and Treatment*.

[8] Podo F., Sardanelli F., Canese R., DAgnolo G. (2002). The Italian multi-centre project on evaluation of MRI and other imaging modalities in early detection of breast cancer in subjects at high genetic risk. *Journal of Experimental & Clinical Cancer Research*.

[9] Morris E. A., Liberman L., Ballon D. J. (2003). MRI of occult breast carcinoma in a high-risk population. *American Journal of Roentgenology*.

[10] Kriege M., Brekelmans C. T. M., Boetes C. (2004). Efficacy of MRI and mammography for breast-cancer screening in women with a familial or genetic predisposition. *The New England Journal of Medicine*.

[11] Warner E., Plewes D. B., Hill K. A. (2004). Surveillance of BRCA1 and BRCA2 mutation carriers with magnetic resonance imaging, ultrasound, mammography, and clinical breast examination. *Journal of the American Medical Association*.

[12] Kuhl C. K., Schrading S., Leutner C. C. (2005). Mammography, breast ultrasound, and magnetic resonance imaging for surveillance of women at high familial risk for breast cancer. *Journal of Clinical Oncology*.

[13] Leach M. O. (2005). Screening with magnetic resonance imaging and mammography of a UK population at high familial risk of breast cancer: A prospective multicentre cohort study (MARIBS). *The Lancet*.

[14] Lehman C. D., Blume J. D., Weatherall P. (2005). Screening women at high risk for breast cancer with mammography and magnetic resonance imaging. *Cancer*.

[15] Lehman C. D., Isaacs C., Schnall M. D. (2007). Cancer yield of mammography, MR, and US in high-risk women: prospective multi-institution breast cancer screening study. *Radiology*.

[16] Wiratkapun C., Duke D., Nordmann A. S. (2008). Indeterminate or suspicious breast lesions detected initially with mr imaging. Value of MRI-directed breast ultrasound. *Academic Radiology*.

[17] Hong M. J., Cha J. H., Kim H. H. (2015). Second-look ultrasonography for MRI-detected suspicious breast lesions in patients with breast cancer. *Ultrasonography*.

[18] Abe H., Schmidt R. A., Shah R. N. (2010). MR-directed ('second-look') ultrasound examination for breast lesions detected initially on MRI: MR and sonographic findings. *American Journal of Roentgenology*.

[19] Youk J. H., Kim E., Kim M. J., Lee J. Y., Oh K. K. (2007). Missed breast cancers at US-guided core needle biopsy: how to reduce them. *RadioGraphics*.

[20] Spick C., Baltzer P. A. T. (2014). Diagnostic utility of second-look US for breast lesions identified at MR imaging: Systematic review and meta-analysis. *Radiology*.

[21] Nakano S., Yoshida M., Fujii K. (2009). Fusion of MRI and sonography image for breast cancer evaluation using real-time virtual sonography with magnetic navigation: First experience. *Japanese Journal of Clinical Oncology*.

[22] Nakano S., Yoshida M., Fujii K. (2012). Real-time virtual sonography, a coordinated sonography and MRI system that uses magnetic navigation, improves the sonographic identification of enhancing lesions on breast MRI. *Ultrasound in Medicine & Biology*.

[23] Nakano S., Kousaka J., Fujii K. (2012). Impact of real-time virtual sonography, a coordinated sonography and MRI system that uses an image fusion technique, on the sonographic evaluation of MRI-detected lesions of the breast in second-look sonography. *Breast Cancer Research and Treatment*.

[24] Fausto A., Casella D., Mantovani L., Giacalone G., Volterrani L. (2012). Clinical value of second-look ultrasound: Is there a way to make it objective?. *European Journal of Radiology*.

[25] Fausto A., Rizzatto G., Preziosa A. (2012). A new method to combine contrast-enhanced magnetic resonance imaging during live ultrasound of the breast using volume navigation technique: A study for evaluating feasibility, accuracy and reproducibility in healthy volunteers. *European Journal of Radiology*.

[26] Fausto Six-year prospective evaluation of second-look US with volume navigation for MRI-detected additional breast lesions. *European Radiology*.

[27] Pons E. P., Azcón F. M., Casas M. C., Meca S. M., Espona J. L. G. (2014). Real-time MRI navigated US: Role in diagnosis and guided biopsy of incidental breast lesions and axillary lymph nodes detected on breast MRI but not on second look US. *European Journal of Radiology*.

[28] Uematsu T., Takahashi K., Nishimura S. (2016). Real-time virtual sonography examination and biopsy for suspicious breast lesions identified on MRI alone. *European Radiology*.

[29] Kang D. K., Jung Y., Han S., Kim J. Y., Kim T. H. (2017). Clinical utility of real-time MR-navigated ultrasound with supine breast MRI for suspicious enhancing lesions not identified on second-look ultrasound. *Ultrasound in Medicine & Biology*.

[30] Aribal E., Tureli D., Kucukkaya F., Kaya H. (2017). Volume navigation technique for ultrasound-guided biopsy of breast lesions detected only at MRI. *American Journal of Roentgenology*.

[31] Park A. Y., Seo B. K., Han H. (2018). Clinical value of real-time ultrasonography-MRI fusion imaging for second-look examination in preoperative breast cancer patients: additional lesion detection and treatment planning. *Clinical Breast Cancer*.

[32] Watanabe R., Ando T., Osawa M. (2017). Second-look US using real-time virtual sonography, a coordinated breast US and MRI system with electromagnetic tracking technology: a pilot study. *Ultrasound in Medicine & Biology*.

[33] Park A. Y., Seo B. K. (2016). Real-time MRI navigated ultrasound for preoperative tumor evaluation in breast cancer patients: Technique and clinical implementation. *Korean Journal of Radiology*.

[34] Guo Y., Sivaramakrishna R., Lu C., Suri J. S., Laxminarayan S. (2006). Breast image registration techniques: a survey. *Medical & Biological Engineering & Computing*.

[35] Rajagopal V., Lee A., Chung J.-H. (2008). Creating individual-specific biomechanical models of the breast for medical image analysis. *Academic Radiology*.

[36] Rizzatto G., Fausto A. (2009). Breast imaging and volume navigation: MR imaging and ultrasound coregistration. *Ultrasound Clinics*.

[37] Siegler P., Holloway C. M. B., Causer P., Thevathasan G., Plewes D. B. (2011). Supine breast MRI. *Journal of Magnetic Resonance Imaging*.

[38] Piron C. A., Causer P., Jong R., Shumak R., Plewes D. B. (2003). A hybrid breast biopsy system combining ultrasound and MRI. *IEEE Transactions on Medical Imaging*.

[39] Causer P. A., Piron C. A., Jong R. A., Plewes D. B. (2008). Preliminary in vivo validation of a dedicated breast MRI and sonographic coregistration imaging system. *American Journal of Roentgenology*.

[40] Ewertsen C., Săftoiu A., Gruionu L. G., Karstrup S., Nielsen M. B. (2013). Real-time image fusion involving diagnostic ultrasound. *American Journal of Roentgenology*.

[41] Mazzei F. G., Gentili F., Guerrini S. (2017). MR lymphangiography: a practical guide to perform it and a brief review of the literature from a technical point of view. *BioMed Research International*.

[42] Gennaro P., Borghini A., Chisci G. (2017). Could MRI visualize the invisible? An Italian single center study comparing magnetic resonance lymphography (MRL), super microsurgery and histology in the identification of lymphatic vessels. *European Review for Medical and Pharmacological Sciences*.

[43] Mazzei M. A., Gentili F., Mazzei F. G. (2017). High-resolution MR lymphangiography for planning lymphaticovenous anastomosis treatment: a single-centre experience. *La Radiologia Medica*.

[44] Moon W. K., Chang J. M., Han W. (2012). Evaluation of tumor extent in breast cancer patients using real-time MR navigated ultrasound: Preliminary study. *European Journal of Radiology*.

[45] Alderliesten T., Loo C., Paape A. (2010). On the feasibility of MRI-guided navigation to demarcate breast cancer for breast-conserving surgery. *Medical Physics*.

[46] Kucukkaya F., Aribal E., Tureli D., Altas H., Kaya H. (2016). Use of a volume navigation technique for combining real-time ultrasound and contrast-enhanced MRI: Accuracy and feasibility of a novel technique for locating breast lesions. *American Journal of Roentgenology*.

[47] Sigurdson L. J., Kirkland S. A. (2006). Breast volume determination in breast hypertrophy: An accurate method using two anthropomorphic measurements. *Plastic and Reconstructive Surgery*.

